# Relationship between spinal imbalance and knee osteoarthritis by using full-body EOS

**DOI:** 10.1186/s12891-023-06508-5

**Published:** 2023-05-19

**Authors:** Pengfei Fu, Wu Xu, Pingcheng Xu, Jun Huang, Jiong Jiong Guo

**Affiliations:** 1 Department of Orthopedics and Sports Medicine, The First Affiliated Hospital of Soochow University, 188 Shizi Street, Suzhou, 215006 People’s Republic of China; 2 Department of Orthopedics, Wujiang Fourth People’s Hospital, Suzhou, People’s Republic of China

**Keywords:** Knee osteoarthritis, EOS imaging system, Sagittal spinopelvic alignment, Low back pain, Patellofemoral joint pain

## Abstract

**Background:**

Orthostatic state is maintained by harmonizing the spine, pelvis and lower extremities. In the past few decades, several studies have demonstrated the associations between spinal imbalance and generalized osteoarthritis. The compensatory mechanisms of pelvis translation and knee flexion, however, have not been fully assessed.

**Methods:**

A total of 213 volunteers, over 40 years of age, were recruited. Radiological measurements were performed by EOS imaging system. Pelvic tilt (PT), pelvic incidence (PI), lumbar lordosis (LL), sagittal vertical axis (SVA), global tilt (GT), hip-knee-angle (HKA), knee flexion angle (KFA), lateral distal femoral angle (LDFA), and medial proximal tibial angle (MPTA) were measured. On the basis of SRS-Schwab, the subjects were classified into decompensated group (PI-LL > 20°), compensated group(10° ≤ PI-LL ≤ 20°), and normal group (PI-LL < 10°). Differences in radiographic parameters among groups were evaluated. Data of Knee Society Score (KSS) and Oswestry Disability Index (ODI) score were collected via questionnaires.

**Results:**

Decompensated group showed larger pelvic parameters (PT) and low extremity parameters (LDFA, MPTA, HKA and KFA) than normal group (*P* < 0.05). Pelvic parameter was larger in the compensated group (median = 31°) compared to the normal group (median = 17°) (*P* < 0.05). There was no difference in low extremity parameters between the compensated and normal groups. At the sagittal plane, the radiological parameters of spine were greater in subjects with patellofemoral joint pain (PFP) than without PFP (*P* = 0.058). Higher PI-LL values were observed in women (*P* < 0.05).

**Conclusions:**

A correlation between sagittal spinal imbalance and knee joint angles was recognized. The progression of knee and low back pain was associated with the severity of sagittal spinal imbalance. Pelvic retroversion was considered to be the probable compensatory mechanism.

**Supplementary Information:**

The online version contains supplementary material available at 10.1186/s12891-023-06508-5.

## Introduction

Bipedal walking is a distinctive feature of the human race. Orthostatic standing state is maintained by harmonizing the spine, pelvis and lower extremities [1–3]. New developments in radiology, such as EOS system, have contributed to huge advances in the comprehension of sagittal spinopelvic alignment in the asymptomatic population and in patients with spinal disorders, hip and knee osteoarthritis. The full-body EOS images provided additional information regarding the global spine and lower extremities alignment to improve the understanding of the patient functional posture [4, 5].

Hip and knee osteoarthritis lead to compensatory joint flexion, lower extremity muscle fatigue and pain, which in turn lead to a deterioration in human health-related quality of life [6]. Low back pain (LBP) also has a serious impact on population’s quality of life. In the past few decades, several studies have demonstrated the associations between spinal imbalance and generalized osteoarthritis, including knee and hip [7–10]. In a Japanese study, alterations in normal spinopelvic parameters led to increased thigh muscle fatigue and knee flexion, which would increase LBP and patellofemoral joint pain (PFP) [11]. Some authors pointed out that spinal symptoms such as lumbar muscle fatigue and LBP, may be caused by degenerative knee disease [12, 13].

The compensatory mechanisms of pelvis translation and knee flexion, however, have not been fully assessed. We hypothesized that changes in spine-pelvis-lower extremity sagittal alignment might lead to LBP, and subsequently cause muscle fatigue, LBP and PFP. The purposes were to analyze the relationship between spinal imbalance and knee osteoarthritis and explore their compensatory mechanisms in subjects with LBP or PFP.

## Materials and methods

### Study subjects

Between Jan 1, 2021 to Mar 31, 2022, three hundred volunteers were recruited to participate in the study. Participants were screened and grouped by two experienced physicians using medical history, physical examination and imaging. Subjects who met the following criteria were included: (1) over 40 years of age; (2) a standing position EOS images of the entire spine and pelvis can be acquired; and (3) informed consent and understanding of this study. Subjects who meet the following criteria will be excluded: (1) presence of deformity pathology (e.g., tumor, infection); (2) underwent arthroplasty on lower extremities (e.g., hip, knee); (3) underwent instrumented spinal surgery; (4) sacralization and lumbarization of the spine; (5) BMI ≥ 25.0 kg/m^2^; (6) the history of hip and spinal pathology.

The subjects were classified into three groups by the SRS-Schwab: group A (PI-LL < 10˚), group B (10˚ ≤ PI-LL ≤ 20˚) and group C (PI-LL > 20˚) [14, 15]. PI-LL < 10° was considered as normal group; 10° ≤ PI-LL ≤ 20°as substitute group; PI-LL > 20°as decompensated group. The selected PI-LL parameter was then sub-stratified based on age into 4 groups: 40–50, 51 to 60, 61 to 70, over 71. Meanwhile, subjects were divided into the PFP group and the control group; the LBP group and the control group, according to the presence or absence of PFP or LBP.

The ethics committee of The First Affiliated Hospital of Soochow University approved the study. Well-informed consent was acquired from each individual and demographic characteristics (age, gender) were collected. Furthermore, participants recruited to our department were asked about the presence and duration of pain in the low back and knee.

### Radiographs and measurements

Radiographic parameters of spinal imbalance patients were collected by using X-ray in most studies, but simple X-ray is not enough to describe the overall level of the spine and the degree of deformity in the lower limbs, with the development of research. Currently, EOS imaging system was the only disposable imaging system in the world.

Using the full-body EOS images (EOS Imaging, Paris, France), we measured the classical spinopelvic parameters to assess the sagittal balance condition (PI, PT, C7 SVA, and GT) and the lower limbs position (HKA angle, KFA, LDFA, MPTA). Measurements of imaging parameters were performed by three independent, trained surgeons. An average of their measurements was recorded.

**Fig. 1:**
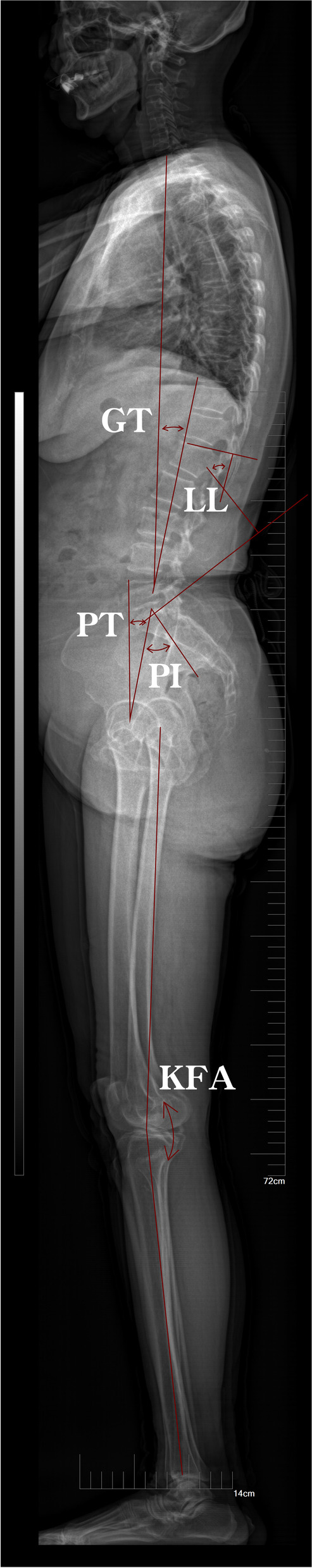
Sagittal parameters in the EOS system. GT: Global tilt; LL: Lumbar lordosis; PT: Pelvic tilt; PI: Pelvic incidence; KFA: Knee flexion angle

**Fig. 2:**
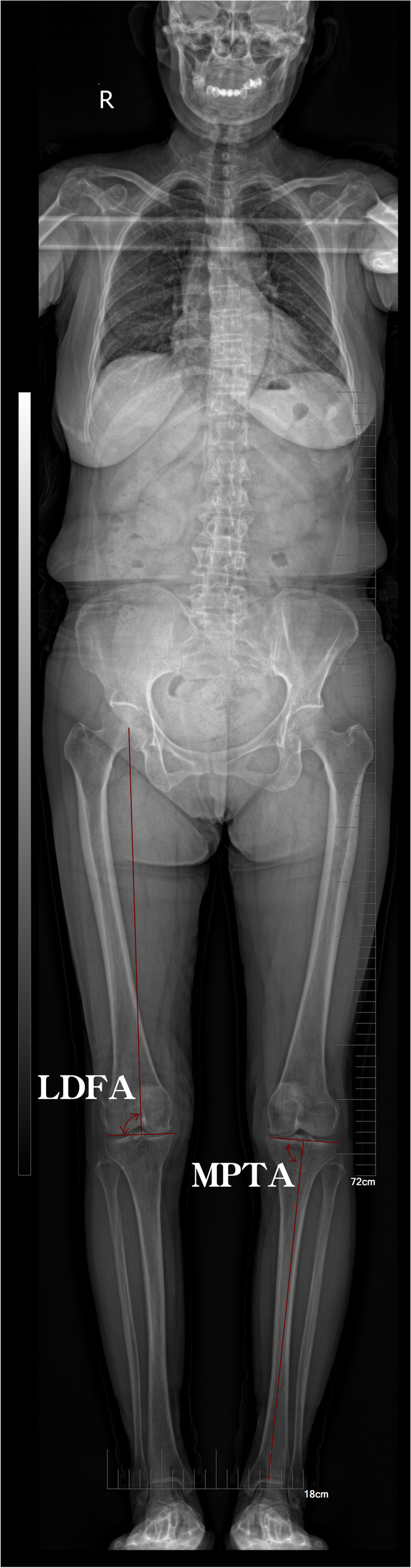
Coronal position parameters in EOS system. LDFA: Lateral distal femoral angle; MPTA: Medial proximal tibial angle

All angular spinal and knee measurements were performed using the Cobb method as per convention in literature (Figs .1 and 2 and Table 1).

**Table 1:** Definitions for Global and Regional Radiographic measurements

Sagittal Vertebral Axis	Horizontal offset distance between an imaginary plumb line dropped from the center of the C7 vertebra to the posterosuperior superior endplate of S1
Pelvic tilt	Acute angle subtended by a line drawn from the midpoint of the superior endplate of S1 to the bicoxafemoral hip center and an imaginary vertical line
Pelvic incidence	Angle subtended by the perpendicular of superior endplate of S1 and a line drawn from the midpoint of the superior endplate of S1 to the bicoxafemoral hip center
Global tilt	Angle subtended by a line drawn from the midpoint of the superior endplate of S1 to the bicoxafemoral hip center and a line from the center of the C7 vertebra to the superior endplate of S1
Lumbar lordosis	Angle measured using the inferior endplate of T12 and the superior endplate of S1
Hip-Knee-Ankle angle	Acute angle between femur mechanical axis and tibial mechanical axis in the coronal plane
Knee flexion angle	Angle between femur mechanical axis and tibial mechanical axis In the sagittal plane
Lateral distal femoral angle	Angle between the femur mechanical axis and the distal articular surface of the femur in the coronal plane
Medial proximal tibial angle	Angle between the tibial mechanical axis and the proximal articular surface of the tibia in the coronal plane

SVA indicates sagittal vertical axis

In addition, the presence of PFP and LBP was assessed by KSS and ODI questionnaires, respectively.

### Data analysis

IBM SPSS statistical software ver. 26.0 (IBM Corp., Armonk, NY, USA) was used for statistical analysis. Pearson correlation was used to characterize linear relationships between radiographic measurements. Comparisons of participant ages and radiographic parameters between subjects’ subgroups were performed using independent sample non-parametric test. The level of significance was defined as *P* ≤ 0.05.

## Results

### Study sample

Three hundred volunteers were recruited, and 213 of which met our inclusion criteria. In total, 52 participants were excluded due to arthroplasty in the hip (*n* = 18) or knee (*n* = 23), or operations on the spine (*n* = 11). 10 participants were excluded because of their BMI (≥ 25.0 kg/m^2^). In addition, further radiographic examination revealed lumbar spondylolisthesis in 14 participants, degenerative lumbar scoliosis in 3 participants, osteoporotic vertebral fracture in 8 participants. Consequently, images were analyzed from the remaining 213 subjects (114 males, 99 females; mean age, 64.4 years, range 40–80).

### Spinopelvic parameters

The median and interquartile range of the studied parameters in the entire study group and either gender group were summarized in Table 2. PI, PT, SVA and GT values were revealed between sex. There was no difference between the two groups in the knee joint angle. However, value of LL was significantly higher in males than in females (49° versus 44°, *p* < 0.05).

**Table 2:** Radiographic parameters in different genders

Variable	SVA(cm)	PT(°)	PI(°)	GT(°)	LL(°)	HKA(°)	KFA(°)	PI-LL(°)	LDFA(°)	MPTA(°)
All	1.9(2)	23(17)	47(23)	23(16)	45(18.5)	2(3)	2(3)	2(26)	88(5)	87(4)
Male	1.9(11.7)	22(21)	46(24)	22(7)	49(20)	2(3)	2(2)	-1(27)	87(4)	87(4)
Female	1.8(12.7)	23(16.3)	48(23.3)	23(16.3)	44(18.3)	1(3)	2(3)	3.5(25.3)	88(5)	87(4)
*p*-value (male:female)	0.697	0.419	0.478	0.475	< 0.05	0.470	0.390	0.088	0.113	0.637

Values are presented as median (interquartile range)

### Schwab classification evaluation of sagittal alignment

The distributions of Schwab classification for male and female subjects were shown in Table 3. Of the entire study group, 139 subjects were classified as Group A, 37 as Group B and 37 as Group C.

**Table 3:** The distribution of Schwab classification evaluation of sagittal alignment

Variable	All	Male	Female
Group A	139(65.2)	68(68.7)	71(62.3)
Group B	37(17.4)	19(19.2)	18(15.8)
Group C	37(17.4)	12(12.1)	25(21.9)
Total	213(100.0)	99(100.0)	114(100.0)

Values are presented as number (%)

Group A:PI-LL < 10 ˚; Group B:10 ˚ ≤ PI-LL ≤ 20 ˚; Group C:PI-LL > 20˚

### Relationships between radiographic parameters

**Fig. 3:**
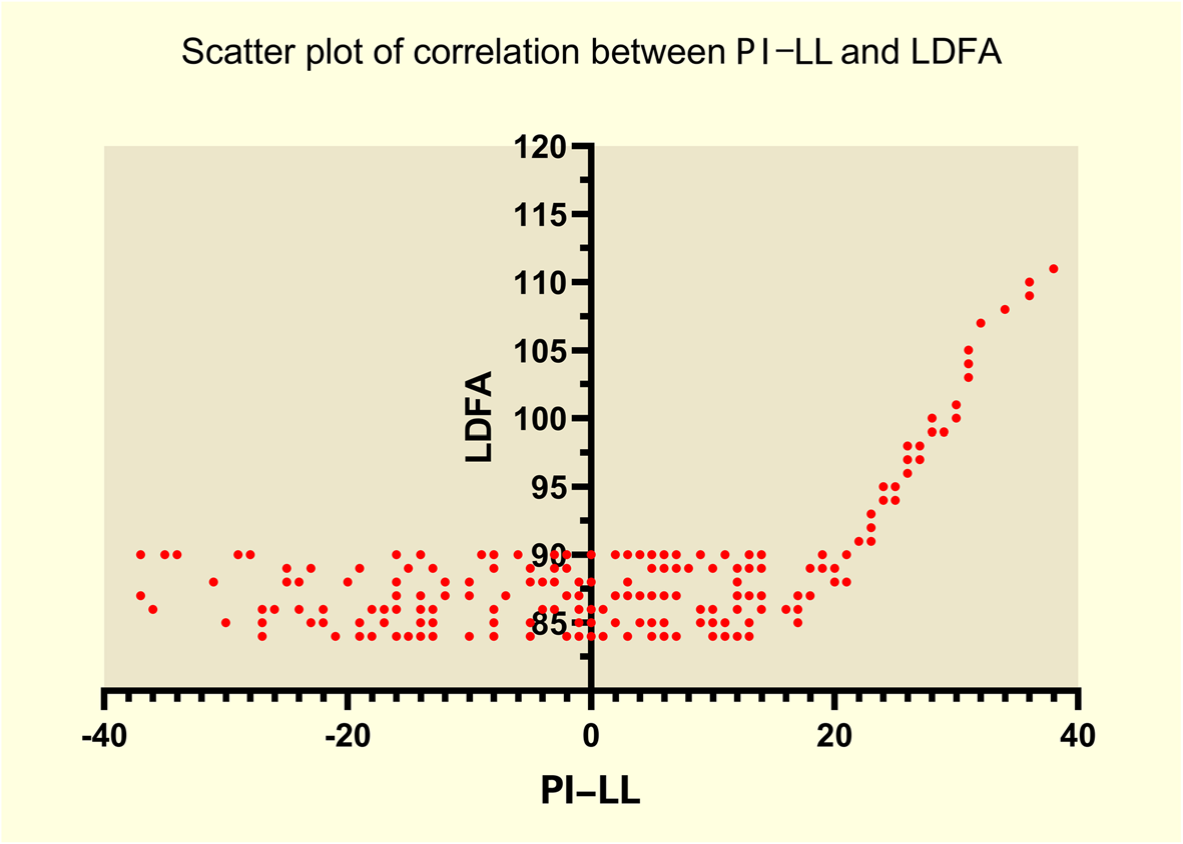
Scatter plot of correlation between PLML and LDFA. The correlation between PI-LL and LDFA. PI-LL: Pelvic incidence minus lumbar lordosis (PI-LL). LDFA: Lateral distal femoral angle

**Fig. 4:**
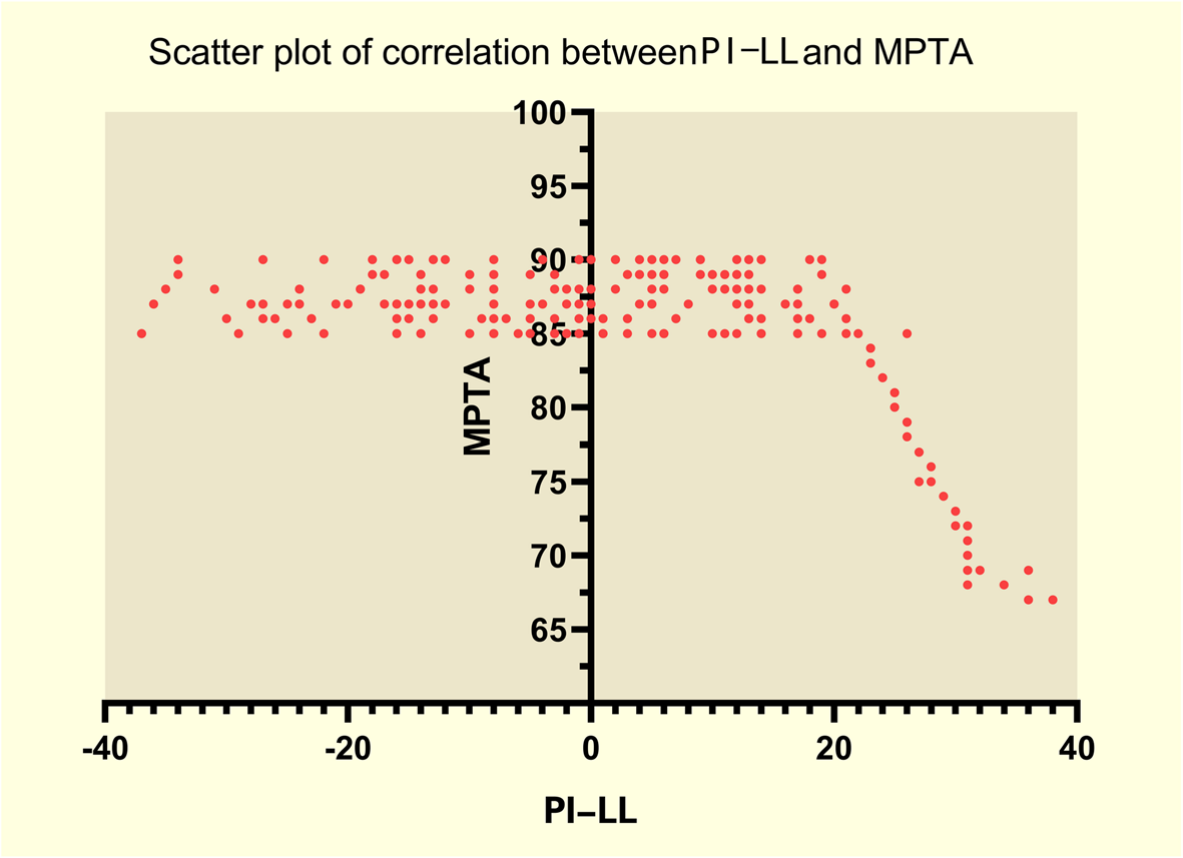
Scatter plot of correlation between PLML and MPTA. The correlation between PI-LL and MPTA. PI-LL: Pelvic incidence minus lumbar lordosis (PI-LL). MPTA: Medial proximal tibial angle

We investigated the linear relationship between PI-LL and knee flexion angle. LDFA tended to be stable when PI-LL was less than 20°, with an average value of 87° (Fig. 3). When PI-LL was greater than 20°, there was a linear correlation with LDFA. MPTA exhibited the same tendency (Fig. 4).

**Fig. 5:**
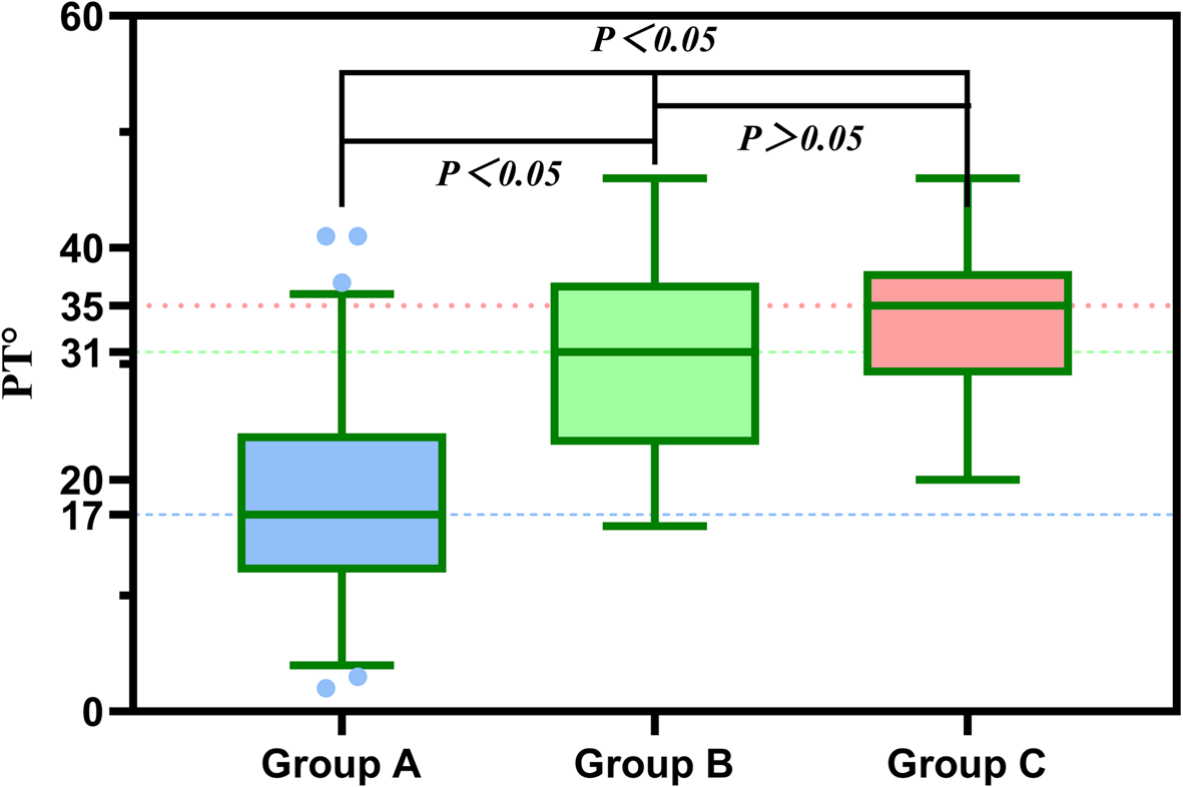
Relationship of PT values between the three groups. Compared with Group A, subjects with PI-LL ≥ 10° (Group B, C) showed larger PT. There was no difference in PT between Group B and Group C

**Fig. 6:**
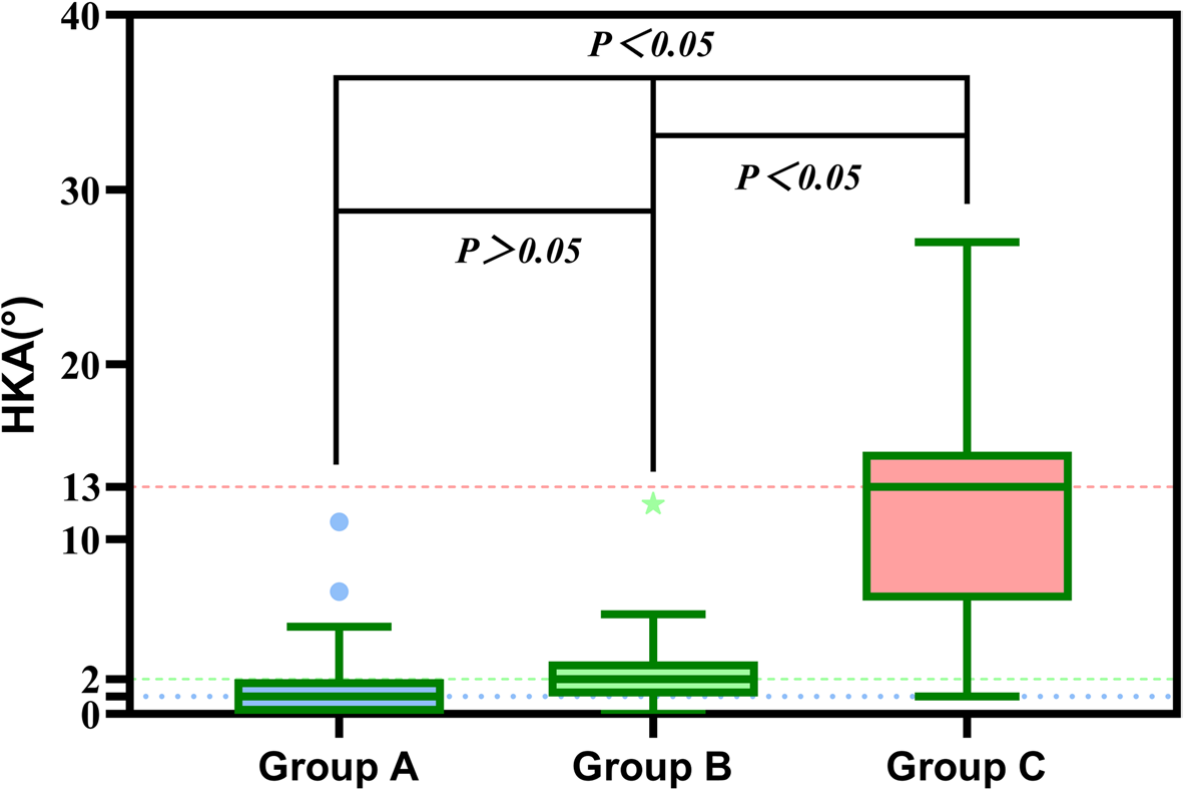
Relationship of HKA values between the three groups. Subjects with PI-LL > 20°(Group C) showed larger HKA value compared with Group A,B. There were no differences in HKA between Group A and Group B

**Fig. 7:**
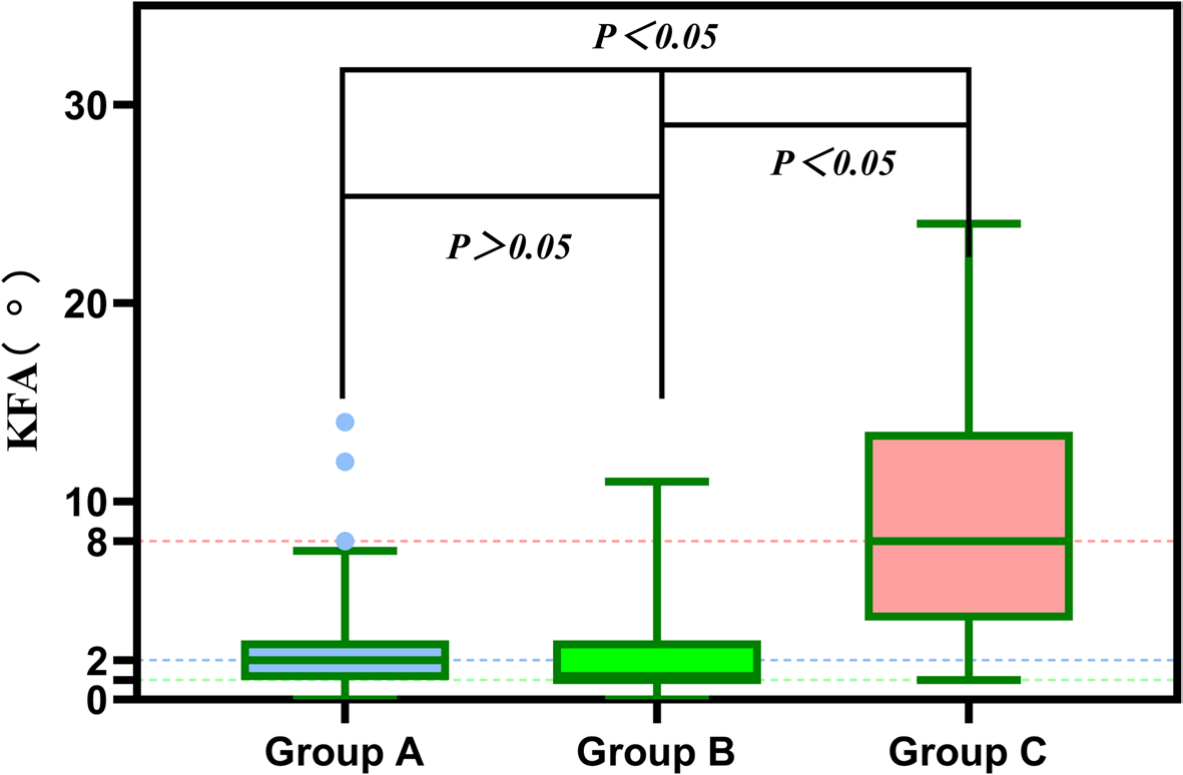
Relationship of KFA values between the three groups. Subjects with PI-LL > 20°(Group C) showed larger KFA value compared with Group A,B. There were no differences in KFA between Group A and Group B

Compared with the normal group, compensated group showed larger PT, but there were no differences in HKA and KFA (Figs. 5, 6 and 7). Conversely, decompensated group showed significantly larger PT values, and larger HKA, KFA values, than the normal group. We found that those with PI-LL > 20° had lager HKA and KFA values than those with PI-LL ≤ 20°. In addition, PT in subjects with PI-LL ≥ 10° was larger than that in subjects with PI-LL < 10°.

### Influence of global and regional spinal sagittal parameters on KSS score and ODI score

**Fig. 8:**
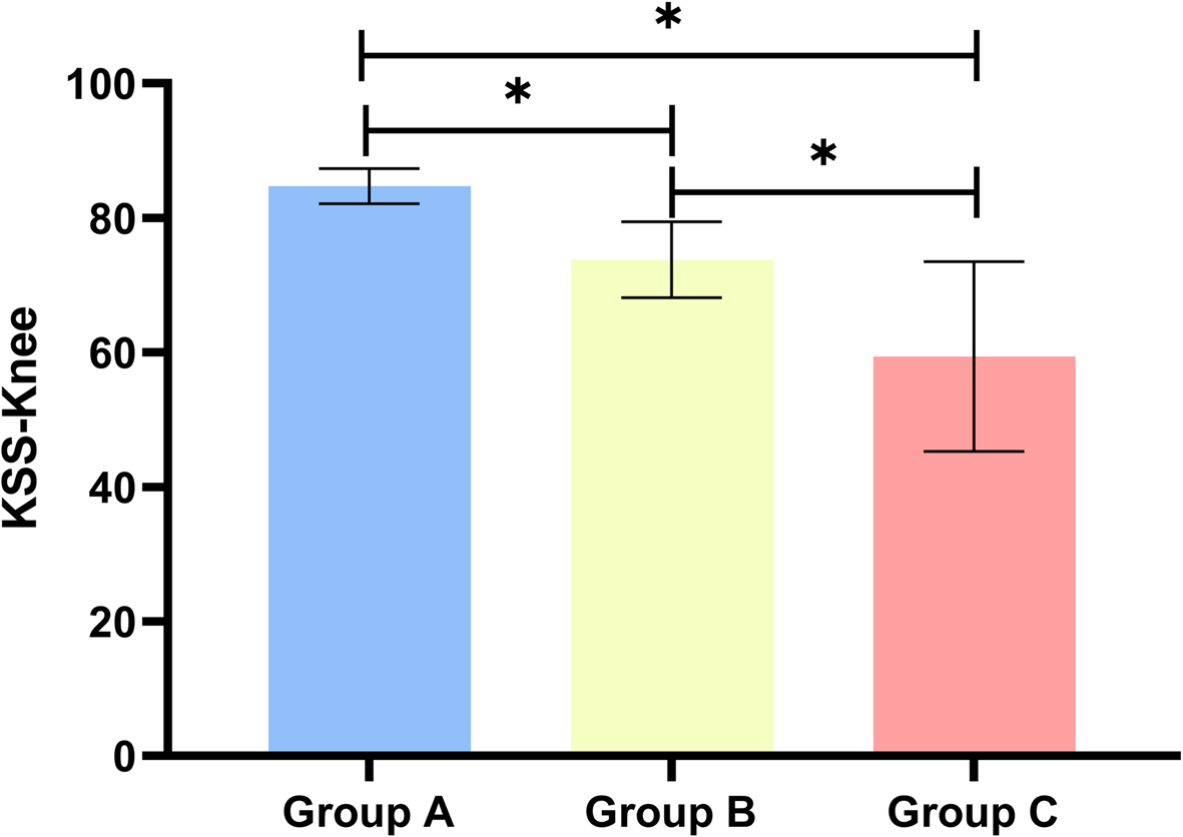
Relationship of KSS scores between the three groups. Histogram showed the mean values for KSS between the three groups

**Fig. 9:**
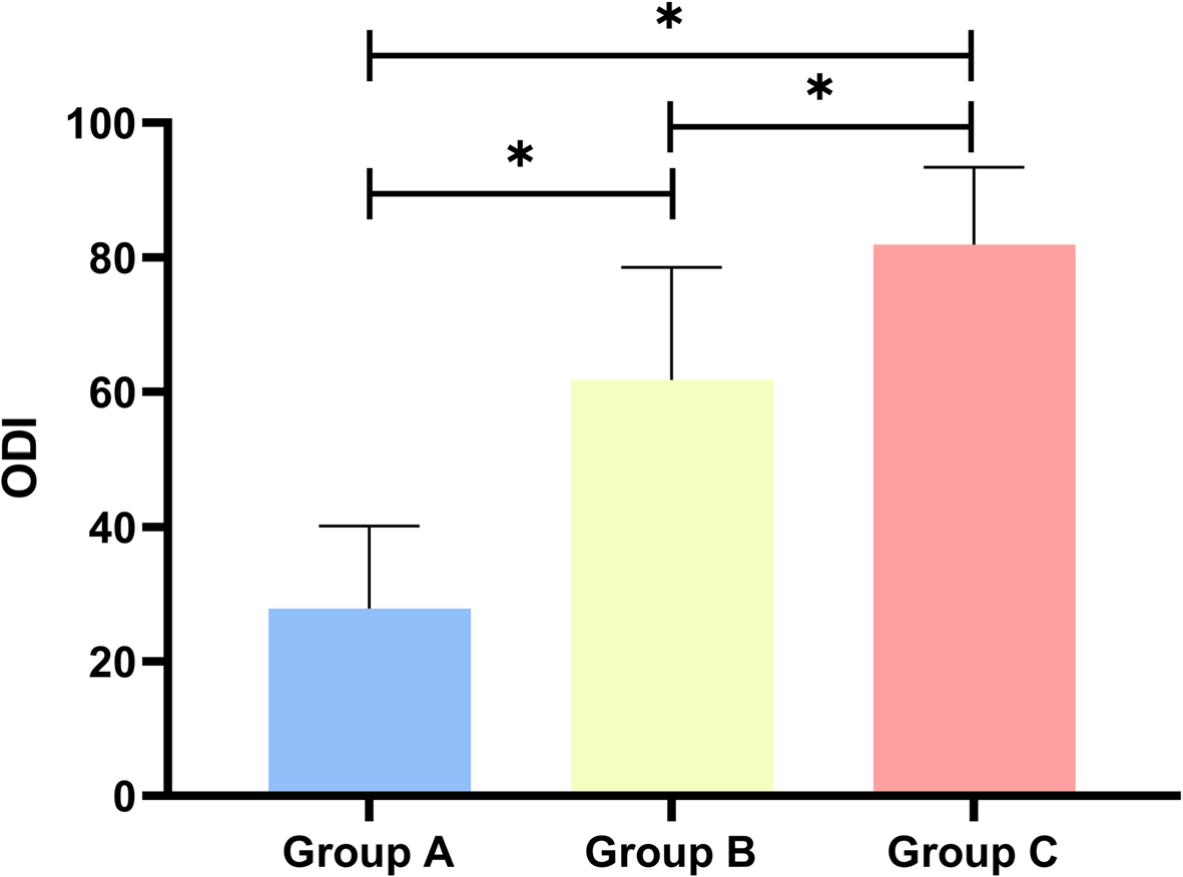
Relationship of ODI scores between the three groups. Histogram showed the ODI scores of low back pain between the three groups

A total of 60 subjects reported PFP. Compared with those without PFP, subjects with PFP presented larger values in almost all sagittal alignment and knee parameters except SVA (Table 4). Similarly, we found that LBP was associated with sagittal alignment and knee parameters (Table 5). Knee score decreased with increasing PI-LL (Fig. 8); whereas ODI score increased with increasing PI-LL (Fig. 9).

**Table 4:** Comparisons of the sagittal spine-pelvis-lower extremity alignment parameters in subjects with or without PFP

	With PFP	Without PFP	*P*-value
SVA	3.4 (8.7–6.9)	1.5 (6.8–4.5)	0.605
GT	32 (35.8–23)	21 (25–14)	< 0.05
T1PA	24 (28–14.5)	11.2 (18–4)	< 0.05
PT	32.5 (37–24)	20 (25.5–13)	< 0.05
PI	61 (67.8–53.8)	44 (54–35)	< 0.05
LL	39 (46.5–24.3)	49 (58.5–41)	< 0.05
PI-LL	21 (26.8–13)	-2 (5–14)	< 0.05
HKA	3 (13–1)	1 (3–0)	< 0.05
KFA	3 (10.8–1)	2 (3–1)	< 0.05
LDFA	90 (98–87.3)	87 (89–85)	< 0.05
MPTA	85 (87–76.5)	87 (89–86)	< 0.05

The comparison was carried out by independent sample non-parametric test. Significant difference in PFP was revealed between the two subgroups of subjects

**Table 5:** Comparisons of the sagittal spine-pelvis-lower extremity alignment parameters in subjects with or without LBP

	With LBP	Without LBP	*P*-value
SVA	8.1 (12.5–1.3)	-1(3.5–6.1)	< 0.05
GT	32 (35–24)	20(25–14)	< 0.05
T1PA	26 (28–17)	19 (21–12)	< 0.05
PT	33 (37–24)	19(25–13)	< 0.05
PI	61 (68–56)	42(53.8–34)	< 0.05
LL	39 (47.5–36)	49(58–40)	< 0.05
PI-LL	21 (27–13)	-2.5(5–14)	< 0.05
HKA	3 (13–1)	1(2–0)	< 0.05
KFA	4 (10.5–1)	2(3–1)	< 0.05
LDFA	90 (98–87)	87(89–85)	< 0.05
MPTA	85 (87.5–76.5)	87(89–86)	< 0.05

The comparison was carried out by independent sample non-parametric test. Significant difference in LBP was revealed between the two subgroups of subjects

### Sex and age in relation to sagittal alignment

**Fig. 10:**
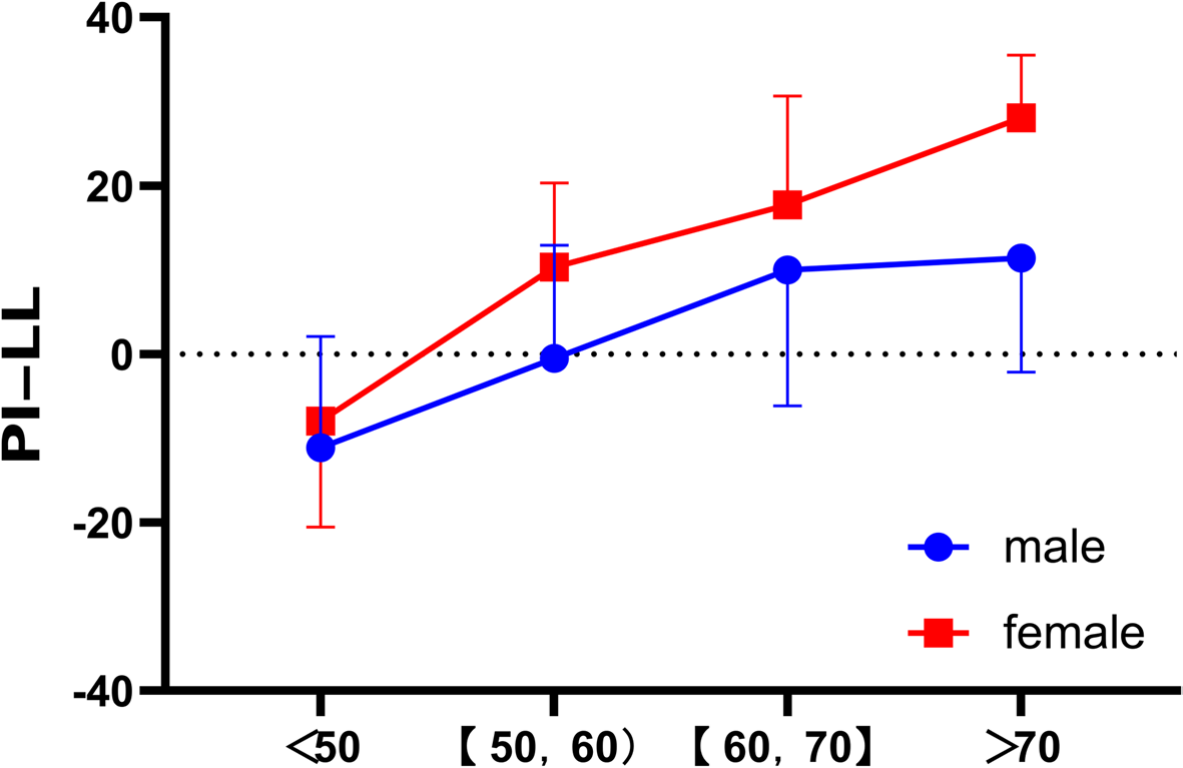
The distribution of PI-LL in male and female. Line showed the distribution of PI-LL in male and female. PI-LL: Pelvic incidence minus lumbar lordosis (PI-LL)

The degree of lumbar deformity was correlated with age. And at any given stage, values of PI-LL in women seem to be higher than in men (Fig. 10).

## Discussion

Sagittal spine-pelvis-lower extremity alignment can be significantly affected by spinal disorders, hip osteoarthritis and knee instability. Abnormal sagittal alignment has been identified as a contributor to the pathogenesis of LBP and PFP. In the present study, a correlation between sagittal spinal imbalance and knee joint angles was recognized. The progression of knee and low back pain was associated with the severity of sagittal spinal imbalance. In addition, pelvic retroversion was considered to be the probable compensatory mechanism.

To date, numerous studies have explored the compensatory mechanisms among spinal deformity, pelvis translation and knee flexion. In a study of 27 patients with severe sagittal imbalance, Ibrahim et al. analyzed the knee flexion angle as a compensatory mechanism for sagittal imbalance [9]. According to Taichi et al. in their study of 399 middle-aged volunteers, decreasing lumbar lordosis led to increasing thigh muscle tension and knee flexion while standing [11]. Conversely, according to a radiographic study, a cross-sectional analysis of 117 individuals with or without severe knee osteoarthritis who underwent x-ray assessment pointed out that the lumbar spine served as the primary source of compensation [12]. Based on the biomechanical analysis of the spinopelvic organization and adaptation in pathology, there is a strong correlation in shape and positioning, and form and function, between the pelvis and the spine [16]. More recently, in a retrospective study of patients with spinal deformity and full-body EOS images, Emmanuelle et al. concluded that patients categorized based on different T1 spinopelvic inclination were found to have significantly different compensatory mechanisms in the pelvic shift and lower-limb [17]. In fact, the authors concluded that forward patients had a small LL, with a large pelvic shift creating compensatory knee flexion. However, mechanisms recruited for this pelvis translation were not assessed.

In our study, we demonstrated that most subjects over 40 years of age with poor lumbopelvic sagittal alignment had severe knee degeneration. To clarify the specific mechanisms of knee degeneration and spinopelvic alignment, subjects were divided into three groups according to the degree of spinal deformity. Compared with the normal group, substitute group showed larger PT but comparable HKA, KFA, LDFA and MPTA, suggesting that the compensation was probably contributed by the pelvic tilt. In contrast, decompensated group showed larger HKA, KFA, LDFA and MPTA, but comparable PT versus substitute group. Therefore, knee flexion is primarily due to a sagittal imbalance that exceeds the compensatory capacity of the pelvis, resulting in increased thigh muscle tension.

Considering that discogenic pain is one of the main symptoms of LBP, reduced lumbar lordosis may be the primary compensatory mechanism for chronic LBP [18–20]. We evaluated the association between radiological parameters and ODI score and found that the ODI score increased with the degree of lumbar deformity. We also confirmed that subjects with PFP are probably to have corresponding sagittal changes in the spine.

Differences in hormone levels, physiology, and lifestyle habits between men and women contribute to the different prevalence of spinal instability. Some studies have indicated that elderly males have a higher prevalence of cervical spondylotic myelopathy [21]. In our study, the incidence of lumbar spine deformity was higher in women versus men. Spinal and pelvic malformations appear to be severe in women of the same age.

Murata et al. found that the loss of lordosis was related to degenerative changes in the knee [13]. In their study, limitation of extension of the knee was significantly greater in patients whose lumbar lordosis was 30˚ or less. These findings indicated that severe spinal deformity tend to be associated with low knee extension. However, in the present study, subjects with severe spinal deformity (PI-LL > 20°) showed posterior pelvic tilt and knee flexion. Notably, the PT was comparable between the subjects with mild and severe imbalance in the sagittal spine, which differed from the previous reported compensation mechanism. We believed that this inconsistency might be a result of the flexibility of the subjects studied. Our study was all middle-aged and elderly individuals. The elderly population may suffer from poor lumbar spine flexibility and limited ability to compensate for knee flexion. This is also likely due to the lack of advanced imaging (EOS) to assess the subtle spine, pelvis and knee changes that may otherwise be missed with plain radiographs or CT. The full-body EOS images provided additional information regarding the global spine and lower extremities alignment to improve the understanding of the patient functional posture [22].

We would like to acknowledge several limitations of our study. Firstly, occupation of the subjects was not recorded in our study. Sedentariness may result in a loss of back extension strength, which may lead to thoracic kyphosis or lumbar lordosis. Secondly, some subjects were individuals with LBP or PFP. It was unknown whether our results were applicable to other groups. Further studies with additional populations are needed. In addition, the alignment analysis in our study focused on the sagittal plane, just like in previous studies. The standing pose is of course in 3D, which may facilitate a comprehensive study of dynamic interactions in spine-hip-lower extremity. Finally, our radiological analysis revealed solely static interactions among sagittal alignment of the spine, pelvic inclination, and knee flexion. Sagittal spine-pelvis-lower extremity alignment is a unified whole. Gait analysis would be useful to understand the dynamic interactions among knee flexion, the pelvis and lumbar spine in daily life [23–25].

Despite these limitations, the relationship between spinal imbalance and knee osteoarthritis was identified in subjects suffering from LBP or PFP. Overall, severe sagittal imbalance of the spine will lead to knee osteoarthritis. The pelvis seemed to serve as the primary source of compensation for disturbances of the sagittal alignment in subjects with mild spinal imbalance (10° ≤ PI-LL ≤ 20°). In subjects with severe spinal imbalance (PI-LL > 20°), the pelvis and knee joint were all involved in compensation, presenting as a posterior pelvic tilt and a flexed knee joint. Our study has further broadened the understanding of mechanisms related to sagittal alignment in adults. Therefore, future research efforts should be devoted to a deeper understanding of the mechanisms involved in different individuals that may contribute to LBP and PFP, with hope to develop preventative or novel therapeutic interventions.

## Conclusion

Based on the cross-sectional analyses of subjects with full-body EOS images, our study recognized a correlation between sagittal spinal imbalance and knee joint angles. Moreover, pelvic retroversion was considered to be the probable compensatory mechanism.

Furthermore, as this study is population-based, it is of tremendous public health importance. There is abundant evidence in the literature demonstrating the strong association of sagittal-plane spinal deformity with hip and knee osteoarthritis. Our current study clearly illustrated that spinal disorders subconsciously affect hip and knee osteoarthritis, and the public should be well -informed that spinal orthopedic is important to prevent hip and knee osteoarthritis. Prevention and treatment of spinal deformity must be a public health priority. If successful, such outcomes might minimize the extent and severity of lower extremity osteoarthritis, which in turn may also reduce the risk of spinal deformity, resulting in a more productive and healthier society.

## Supplementary Information

**Additional file 1: Fig. S1.** The flowchart of the exclusion criteria. **Fig. S2**. EOS can obtain accurate 2D and 3D data. **Fig. S3.** The most of radiological parameters are presented in the EOS report.

## Data Availability

The datasets generated and/or analyzed during the current study are not publicly available due to limitations of ethical approval involving the patient data and anonymity but are available from the corresponding author on reasonable request.

## Abbreviations

PT: Pelvic tilt
PI: Pelvic incidence
LL: Lumbar lordosis
SVA: Sagittal vertical axis
GT: Global tilt
T1PA: T1 pelvic angle
HKA: Hip-knee-angle
KFA: Knee flexion angle
LDFA: Lateral distal femoral angle
MPTA: Medial proximal tibial angle
PFP: Patellofemoral joint pain
LBP: Low back pain
PI-LL: Pelvic incidence minus lumbar lordosis
