# Supplementary material for: Relationship between spinal imbalance and knee osteoarthritis by using full-body EOS
Source: BMC Musculoskelet Disord. 2023 May 19;24:402. doi: 10.1186/s12891-023-06508-5 (PMC10197256; doi:10.1186/s12891-023-06508-5)
Supplement: Supplementary file 1 — Additional file 1: Fig. S1. The flowchart of the exclusion criteria. Fig. S2. EOS can obtain accurate 2D and 3D data. Fig. S3. The most of radiological parameters are presented in the EOS report. [file 12891_2023_6508_MOESM1_ESM.pdf]

Fig.s1

Title：The flowchart of the exclusion criteria

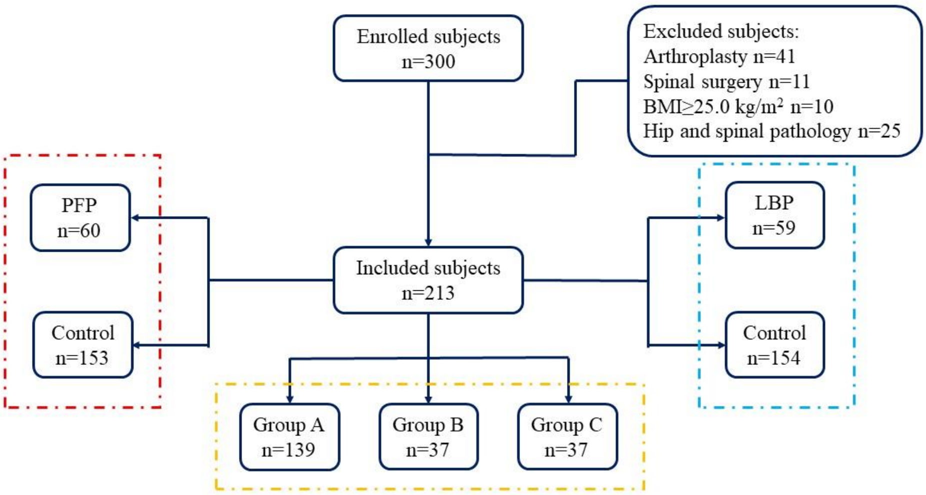

Fig.s1 300 volunteers were recruited, and 87 of which were excluded. The remaining 213 subjects were divided into three large groups according to the needs of the experiment.

PFP: patellofemoral joint pain
LBP: Low back pain

Fig.s2

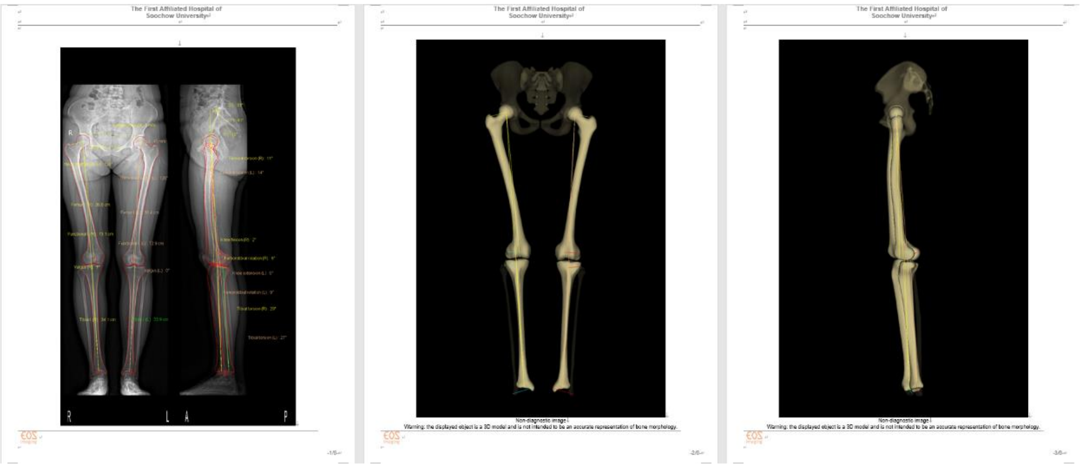

Fig.s2 EOS can obtain accurate 2D and 3D data.

Fig.s3

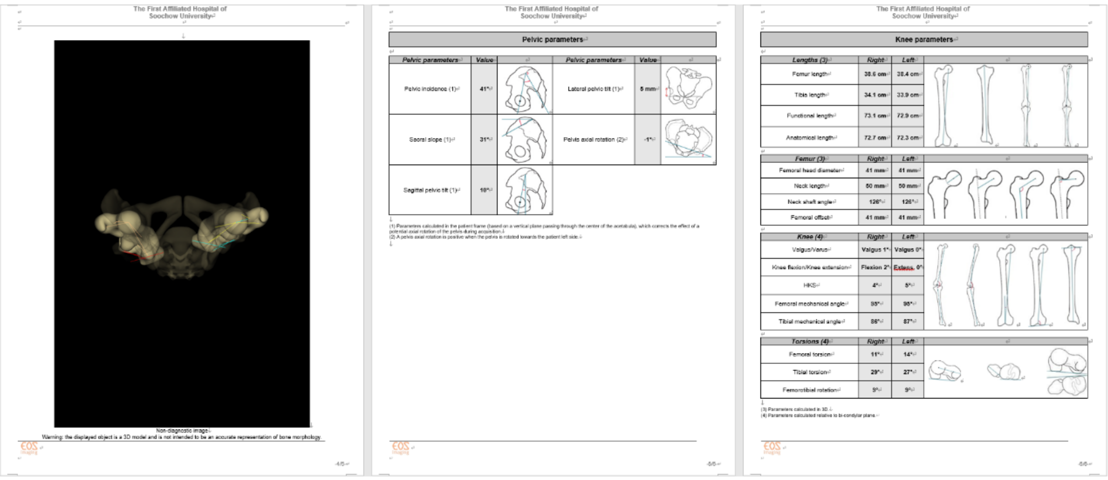

Fig.s3 The most of radiological parameters are presented in the EOS report..
